# Direct and indirect exposure to violence and psychological distress among civil servants in Rio de Janeiro, Brazil: a prospective cohort study

**DOI:** 10.1186/s12888-015-0487-9

**Published:** 2015-05-07

**Authors:** Claudia S Lopes, Claudia L Moraes, Washington L Junger, Guilherme L Werneck, Antonio C Ponce de Leon, Eduardo Faerstein

**Affiliations:** 1 Department of Epidemiology, Institute of Social Medicine, State University of Rio de Janeiro, Rua São Francisco Xavier 524 / 7017D, 7° floor, Rio de Janeiro, CEP 20550-013 RJ Brazil; 2 Department of Public Health Sciences, Division of Social Medicine, Karolinska Institutet, 171 77 Stockholm, Sweden

**Keywords:** Brazil, Mental health, Psychological distress, Violence exposure, Community violence, Cohort study

## Abstract

**Background:**

Important social and economic changes accompanying the recent fast rate of urbanization have been considered a major factor in triggering and sustaining urban violence in Brazil. The purpose of this paper is to investigate the effects of exposure to direct, indirect, and contextual violence on the risk of psychological distress.

**Methods:**

Prospective longitudinal study carried out among 3,058 civil servants working at university campuses in Rio de Janeiro. Psychological distress was measured using the General Health Questionnaire, and exposure to individual violence was assessed as direct (DV), indirect (IV), and both direct and indirect (DIV). Contextual violence was assessed through the geocoding of residential addresses of study participants and the rates of homicides in 2005 at the corresponding weighting area. Multiple logistic regression was used to evaluate individual and contextual correlates of psychological distress.

**Results:**

Exposure to DIV increased more than six times (95% CI 2.7–16.0) the odds of psychological distress occurrence at the six-year follow-up. Regarding persistence of psychological distress, the association with violence exposure was 1.6 (95% CI 1.0–2.4) for DV and 2.7 (95% CI 1.3–5.3) for IV. Contextual violence was not associated with psychological distress, and no interaction effect was found between exposure to individual and contextual violence in the occurrence/persistence of psychological distress.

**Conclusions:**

Results of this study highlight the importance of assessing multiple forms of violence in research on the social determinants of mental disorders and support the view that individual exposure to different forms of violence increases the risk of psychological distress.

## Background

In recent decades, Brazil has faced major social, economic, and demographic changes. Data from Brazilian Institute of Geography and Statistics (IBGE), which is responsible for the official census of Brazil, show that from 1950 to 2010 the population increased from 52 million to 190 million people, with a rate of urbanization rising from 40% to 80%. The fast rate of urbanization led to the concentration of the population in large metropolitan areas, contributing to the organization of a pool of urban workers and the emergence of a sizeable middle class. Important social and economic changes accompanied this demographic transition, and it is considered a major factor in triggering and sustaining urban violence. Thus, an increase in violence has been associated with factors such as rapid urbanization with consequent concentration of the population in big cities, inequality in wealth distribution, changes in employment relationships, greater competition and impersonality in social relationships, and a reduction in traditional networks of social support. In addition, greater access to firearms, police violence, drug trafficking, and environmental degradation are intermediate factors contributing to the increase in urban violence [1,2].

A report from the United Nations Office on Drugs and Crime (UNODC), [3] Global Study on Homicide, estimates that the total number of annual deaths due to homicides in 2010 was 468,000. From those, 31% occurred in the Americas. In the sub-regions in South America, 74% of homicides are committed with a firearm [3]. Data from the Pan American Health Organization [4] show that in the period 2000–2007, the average rate of homicides in the Americas was 17.8 per 100,000, and that in Brazil the rate was the highest in the Americas, reaching 31.0 per 100,000 inhabitants.

In Rio de Janeiro, these data seem even more alarming. The proportion of homicides among external causes of death, increased from 33.4% in 1980 to 45.2% at the end of 1988. This escalation continued in the 1990s, and in 1995, the number of deaths in Rio de Janeiro from external causes exceeded 800 deaths per month [5]. In 2004, data from the Municipal Health Secretariat of Rio de Janeiro (SMS-RJ) showed that the number of deaths from violent attacks was over 3,000. As a result of early deaths associated with high homicide rates, homicide was the leading cause of Potential Years of Life Lost (PYLL) in 1990, accounting for 18.2% of all PYLL in Rio de Janeiro [6]. More recent data from the SMS-RJ show that among men, the number of years of life lost due to violent causes was 3.54 years in 2005. In recent years, a declining trend in deaths due to community violence has been recorded in official statistics, but these statistics have not been compiled over a period long enough to detecting a trend of steady decline.

### Violence, context, and mental health

Violence has been identified as one of the most important stressful life events associated with mental disorders in developed countries [7-9]. In Brazil, despite the escalation of violence in recent decades, the literature on this subject is still relatively scarce, and most of the studies have evaluated the role of family violence in physical and mental health. A study conducted among children and adolescent (7–14 years) from Brasilia (the Brazilian capital) showed that family violence was strongly associated with higher rates of psychiatric disorders [10]. A more recent population-based household survey conducted among women in Sao Paulo and Zona da Mata in Pernambuco found a significantly higher prevalence of mental disorders among women who had experienced family violence than among those who had not [11].

We identified only a few studies in Brazil that evaluated the association between community-violence and mental disorders. Three of them were population-based surveys in which violence was assessed in the context of other stressful life events (SLE), and two studies assessed violence in a more specific way. All population-based studies found correlations between exposure to traumatic events and mental disorders. A study conducted in the late 1990s in Pelotas, southern Brazil, showed that exposure to violence contributed to a higher prevalence and persistence of psychological distress [12] and two recent studies showed similar results. A study carried out in São Paulo showed that out of seven crime-related traumatic events examined, six were associated with at least one psychiatric disorder [13]. Another recent study conducted in São Paulo and Rio de Janeiro found that traumatic events correlated with all psychiatric diagnoses, being assaultive violence correlated with alcohol dependence and depression [14]. The two other studies identified were conducted among civil servants of a public university in Rio de Janeiro. These studies found cross-sectional and prospective evidence regarding the effect of physical aggression and assault or robbery by means of violence on the incidence and the prevalence of common mental disorders [15,16]. The latter also showed that experiencing physical violence in two different occasions about two years apart was associated with a higher risk of psychological distress, suggesting a cumulative effect [16].

Although the majority of studies on the correlates of mental disorders have largely focused on individual factors, there is a growing number of studies that sought to evaluate the impact of community violence exposure on health, considering not only individual exposure, but also factors related to their urban environment (contextual exposure). Poverty, environmental degradation, and other local characteristics and their association with violence have been studied in several countries. Apart from some evidence that support the hypothesis that contextual characteristics from neighbourhood may not affect the probability of violence, for many authors, the underlying assumption is that the neighbourhood organization and social characteristics explain most of the variation in criminality and violence, which may not be attributable only to the characteristics of the individuals. Thus, the ability of certain areas to sharing common values and maintaining social control accounts for the variations in the violence rates observed in different communities [17-20].

The international literature on the role of community or contextual violence in mental disorders is scarce. Most studies investigated this association in the youth and regarded conduct disorders and substance abuse as the main outcomes [21-23]. Other studies investigating victimization in general have examined the relation between armed conflicts and terrorism and the occurrence of post-traumatic stress disorder [24-26]. Moreover, nearly all studies are cross-sectional and unable to ascertain the role of exposure to violence in the risk of developing mental disorders.

Even though Rio de Janeiro is a city with high rates of morbidity and mortality due to external causes, and in particular the various forms of violence, this is the first study using longitudinal data on both contextual and individual violence and mental health in a population living in it. To our knowledge, the present study is also the first one to assess the role of three different domains of violence: direct, indirect (witnessing violence), and contextual violence, in the occurrence and persistence of mental disorders.

We report longitudinal results from the Pró-Saúde Study, a prospective assessment of the influence of psychosocial factors on health among non-faculty civil servants in a university in Rio de Janeiro, Brazil. The aims of this study are (i) to examine the effects of direct violence (suffering physical aggression, being mugged or robbed, being injured with a weapon), indirect violence (witnessing a person being injured or killed with a weapon), and both forms of victimization on the occurrence and persistence of psychological distress six years later; (ii) to investigate the effects of contextual violence (by means of the rate of homicides by area of residence) on the risk of psychological distress; and (iii) to evaluate the potential interaction between contextual and individual violence on the occurrence/persistence of psychological distress.

## Methods

### Design and study population

The Pró-Saúde Study was set up in 1999 to investigate, in a prospective longitudinal study, the degree and causes of the social gradient in physical and mental morbidity. The target population were 4,614 non-faculty civil servants aged 18–65 years and working at a university in Rio de Janeiro. Except for those transferred to another institution or who were on non-health leave (n = 155), all employees were invited to participate, comprising the eligible population for the study (n = 4,459 subjects). With a participation rate of 90.4%, 4,030 men and women were enrolled and completed a self-administered questionnaire in 1999 (phase 1). Further data collections were carried out in 2001 (phase 2) and 2006/2007 (phase 3). Herein, phase 2 will be referred to as the baseline measurement and phase 3 as the follow up. Out of the 4,030 respondents who participated in phase 1, 3,253 (81%) participated in phase 2 and 3,058 (76%) in phase 3.

### Measures

Self-administered questionnaires were completed at the workplace and utilised at all phases to collect information relating to socio-demographic characteristics, family history, work environment, history of stressful life events including exposure to violence and discrimination, medical history, self-reported health, psychosocial and lifestyle factors, social network and support, alcohol consumption, and psychological distress. Diverse methods to improve the quality of the data were applied, including pilot studies, validation of scales, and reliability testing.

#### Outcome measure: psychological distress

Psychological distress was evaluated at all phases of the study by means of the Brazilian version of the General Health Questionnaire-12 items (GHQ-12) [27,28]. Scores for individual items were coded as absent or present (0 or 1) and then added, and those whose total scores were 3 or more (out of 12) were classified as cases [29]. In Brazil, the GHQ-12 was validated against a structured psychiatric interview, and a similar criterion of 3 or more for cases of psychological distress was identified with a sensitivity of 85% and a specificity of 79% [28]. More recently, a study conducted among a community sample of Chinese living in Brazil found that this cut-off point (3 or more) yielded a sensitivity of 75% and specificity of 71% [30].

#### Exposure measurement: individual and contextual violence

##### Individual violence exposure

Physical violence experience was measured at the baseline by means of the following questions: (1) “In the last twelve months, have you been a victim of assault or robbery by means of violence?”; (2) “In the last twelve months, have you been a victim of physical aggression?”; (3) “In the last twelve months, have you been injured with a firearm (handgun, shotgun, pistol, etc.) or weapon (knife, razor, etc.)?” Witnessing violence was assessed by the question “In the last twelve months, have you witnessed someone being injured with a firearm (handgun, shotgun, pistol, etc.) or weapon (knife, razor, etc.)?”.

Questions about violence exposure were aggregated in one variable called “victimization,” which was categorized as follows: (0) no exposure to any type of violence; (1) exposure only to direct violence (DV) (assault or robbery, physical aggression, harmed by a firearm); (2) exposure only to indirect violence (IV) (witness); and (3) exposure to both direct and indirect violence (DIV).

##### Contextual violence

In this study, contextual violence was assessed through the geocoding of residential addresses of study participants and the rates of homicides in 2005 at the corresponding weighting area. Weighting area is a geographical unity formed by contiguous and mutually exclusive census tracts used to calibrate the sampling weights. Weighting areas representing more homogeneous units, in which census sectors considered subnormal – favela areas – were grouped in specific units, nonetheless representing the boundaries of neighbourhoods and administrative regions in the municipality. This grouping strategy is particularly important for Rio de Janeiro, given the heterogeneity of this city’s social space [31]. This allowed us to incorporate the contextual level in the analysis models. The counts of homicides victims in each area were obtained by geocoding the address registered on the death certificates, using every available source of information, that is, a street map database, Google Maps, and street directory.

#### Covariates

The covariates regarded for modelling were age, sex, income, education, and alcohol consumption.

Per capita monthly income was calculated as total family income divided by the number of family members living on that income and categorized into three levels (lower, intermediate, and upper) by using tertiles as cut-off points. Education was measured using the Brazilian educational system and categorized in three levels: elementary (up to 6 years of study), secondary (up to 12 years), and higher (more than 12 years).

Information on the amount and frequency of alcohol intake was obtained by means of three questions. Participants were first asked if they had consumed any type of alcoholic beverage in the last two weeks. If the answer was “yes,” then two other questions were asked: “In the last two weeks, how many days did you drink any type of alcoholic beverage?” and “In the last two weeks, on the days you drank alcohol, in general, how many alcoholic beverages did you drink? Thus, alcohol consumption was defined as: no consumption in the last two weeks, or for those who consumed any alcohol in the last two weeks, mild, moderate, or heavy, according to the number of days of alcohol consumption and alcoholic drinks consumed.

### Statistical analysis

The year 2001 was considered as the baseline and 2006/2007 as the 6-year follow-up evaluation. We analysed the data of the 3,058 subjects who participated in the two phases of the study.

In order to assess the role of exposure to violence on psychological distress, separate analyses were carried out for occurrence (proportion of non-cases at baseline that became cases at follow-up) and persistence (proportion of cases at baseline that were also cases at follow-up). Based on previous literature, the potential confounders age, sex, income, education, and alcohol consumption were included in the multivariate logistic regression models.

The effect of contextual violence was assessed by means of multilevel logistic regression models in which the rate of homicides at each weighting area was a second-level variable. It was also tested the interaction effect between the rate of homicides and the aforementioned individual characteristics.

Odds ratios (OR) and 95% confidence intervals (95% CI) for the occurrence and persistence of psychological distress at the 6-year follow-up were estimated by using logistic regression models for those exposed to DV, IV, and DIV compared to those unexposed at the baseline. Odds ratios of the interaction effect were computed based on the sum of coefficients of the exposure categories and the interaction term. Total variance was also estimated in order to compute confidence intervals for the interaction effects.

### Ethical considerations

The research protocol was approved by the Ethics Committee of the Institute of Social Medicine of the State University of Rio de Janeiro, including questionnaires, recruitment protocol, and informed consents. The participation in the research was voluntary and written informed consent was obtained from all participants at all phases of the cohort study and their privacy assured. At all stages of data collection, the questionnaires were identified only by ID numbers, and their connection to the employee's names was confidential.”

Since questioning about exposure to violence in a survey is a sensitive issue, we took an additional series of precautions to minimize any risk for the participants. First, the questionnaire was self-administered, avoiding direct contact between interviewers and participants. Second, no specific questions on intimate partner violence were included in the questionnaire. Third, all field workers were trained to identify physical or mental distress situations and inform participants that, if they need, they could be immediately referred to a University health care facility.

## Results

Overall, the prevalence of psychological distress was 33.6% at baseline (2001). In 2006/2007, the proportion of occurrence of psychological distress was 23.9% and the proportion of persistence was 59.4%. The risk of being a case in 2006/2007 was 5.2 times (95% CI; 4.4–6.1) greater for those who had been identified as a case at baseline as compared with non-cases. The prevalence of exposure to violence at the baseline (2001) was 11.3% for DV, 3.8% for IV, and 1.8% for DIV.

Psychological distress in 2006/2007 was associated with being a woman (p < 0.0001), having a low per capita income (p < 0.0001), and being a heavy alcohol consumer (p=0.03). Age and education were not statistically associated with presence of psychological distress at follow-up (2006/2007) (Table 1).

**Table 1:** **Psychological distress at follow-up according to demographic characteristics of the study population and alcohol consumption** ^**+**^

		Psychological distress	
		N (%)	p-value ^ * ^
Sex	Male	401 (30.4)	< 0.0001
	Female	688 (40.7)	
Age (years)	≤29	71 (40.8)	0.34
	30-39	366 (34.6)	
	40-49	463 (37.2)	
	≥50	189 (35.7)	
Education	Elementary or less	428 (34.6)	0.12
	High school	380 (36.0)	
	College or more	259 (39.4)	
Household *per capita* income^**^	Lower	381 (41.6)	<0.0001
	Intermediate	312 (38.0)	
	Upper	316 (30.4)	
Alcohol consumption	No consumption	510 (36.1)	0.03
	Mild or moderate	465 (35.7)	
	Heavy	68 (46.9)	

^*^p-value based on Chi-Square test.

^**^tertiles.

^**+**^Pró-Saúde Study (2001–2007), Rio de Janeiro, RJ, Brazil.

Past six years, the frequency of occurrence of psychological distress among those who had been exposed to DV, IV, and DIV at baseline was 28.2%, 23.0%, and 59.1%, respectively. Frequency of persistence of psychological distress was 69.4% for exposure to DV, 76.9% for IV, and 57.6% for DIV. After adjusting for sex, age, education, per capita household income, and alcohol consumption, no statistically significant association was found between being exposed to either DV or IV and odds of psychological distress occurrence, but those exposed to DIV presented a more than six-fold increased odds of occurrence of psychological distress (OR=6.5; 95% CI 2.7–16.0) (Table 2). As for the odds of persistence of psychological distress, the association with violence exposure was 1.6 (95% CI 1.0–2.4) for DV and 2.7 (95% CI 1.3–5.3) for IV; no statistically significant association was found for DIV (Table 2).

**Table 2:** **Occurrence and persistence of psychological distress according to exposure to violence and odds ratios** ^**+**^

	Occurrence of psychological distress			Persistence of psychological distress		
Violence exposure	N (%) ^ * ^	Unadjusted OR (95% CI)	Adjusted OR (95% CI) ^ *** ^	N (%) ^ ** ^	Unadjusted OR (95% CI)	Adjusted OR (95% CI) ^ *** ^
Not exposed	384 (22.7)	1.00	1.00	454 (56.9)	1.00	1.00
Direct	55 (28.2)	1.34 (0.96-1.86)	1.31 (0.92-1.85)	93 (69.4)	1.72 (1.16-2.55)	1.57 (1.02-2.40)
Indirect	14 (23.0)	1.01 (0.55-1.86)	0.92 (0.48-1.78)	40 (76.9)	2.53 (1.31-4.89)	2.65 (1.33-5.28)
Direct/Indirect	13 (59.1)	4.92 (2.09-11.59)	6.52 (2.65-16.04)	19 (57.6)	1.03 (0.51-2.08)	1.07 (0.49-2.36)

^*^p < 0.0001.

^**^p=0.003.

^***^Based on multivariate logistic regression analysis controlling for sex, age, education, *per capita* household income, and alcohol consumption.

^+^Pró-Saúde Study (2001–2007), Rio de Janeiro, RJ, Brazil.

OR: odds ratio; CI: confidence interval.

There was no significant association between rate of homicides and psychological distress at the contextual level by neither the multilevel nor the interaction analysis.

## Discussion

To our knowledge, this is the first longitudinal study on the association of multiple domains of violence exposure (direct, indirect, and contextual violence) in the occurrence and persistence of mental disorders. Moreover, this is one of the few studies assessing violence exposure and mental disorders in a large sample of civil servants living in a metropolitan area from a middle-income country.

Individuals exposed to DIV presented more than six-fold increased odds of psychological distress occurrence at the six-year follow-up; for persistence of psychological distress over this period, exposure to DV was associated with nearly two-fold increased odds, whereas for IV exposure this odds was 2.7 times greater. These results support the hypothesis that violence exposure is associated with increased risk of psychological distress. Since no other study has evaluated exposure to violence in the same way and only a few have used longitudinal data, these findings do add to the existing literature.

Prior cross-sectional investigations of the effects of violence on mental disorders or mental health symptoms have also shown consistent results and established the basis for longitudinal investigations [32-36,14]. Some variations across studies may be due in part to differences in the measures used to define violence exposure. Our findings concur with Hedtke et al. [37], who using a household sample of 4,008 adult women found that lifetime violence exposure was associated with increased risk of depression and PTSD, and the odds of occurrence of these disorders increased with the number of different types of violence experienced, including witnessed violence. A study conducted among current and former drug users (n=786) nested in 270 block groups within Baltimore, Maryland, USA also found longitudinal evidence of the association between exposure to violence and levels of psychological distress [38].

Our study also demonstrated that participants who witness violence are at increased risk of persistence of psychological distress. Hypotheses regarding the relationship between witnessed violence and mental health outcomes have been supported in the literature. A population-based study, conducted with adolescents from a 2005 US national household sample, showed that those exposed to multiple incidents of witnessed community violence were 2.2 times more likely to have a diagnosis of major depression and 2.7 times more likely to have a diagnosis of posttraumatic stress disorder [39]. Another study conducted among women living in urban neighbourhoods in the north-eastern United States showed that those who witnessed violent acts in their neighbourhood were twice as likely to have depressive and anxiety symptoms compared to those who did not witness community violence [40].

When we look at the results as a whole, some points draw attention and deserve to be discussed. Our findings show a robust association between exposure to both direct and indirect violence and the occurrence of psychological distress. However, the association between exposure to indirect violence (IV) and persistence of psychological distress was stronger than exposure to direct violence (DV). There is evidence that exposure to both indirect and direct violence may independently affect mental health [40-44]. It is possible that exposure to both direct and indirect violence (DIV) has a stronger effect on triggering mental health problems (as implied by the 6-fold increase in their occurrence), but day-by-day experience of witnessing violence may play a more important role in maintaining such problems. Concerning the lack of association between DIV and “persistence” whilst it was associated with DV and IV, we acknowledge that it is a counter-intuitive finding. Since violence was measured only at the baseline, violence events that may have occurred between 2001 and 2006 were not accounted for. For instance, it is possible that those exposed to DIV at the baseline have not experienced any violent event during the follow-up window. Hence, if persistence of psychological distress needs a continuous exposure to stressful life events, this might explain our findings. Further studies should be carried out in order to clarify these issues.

Our results did not support the hypothesis that higher rates of homicides at the contextual level would increase the risk of psychological distress occurrence or persistence. Prior research on the contextual effect of violence on the risk of mental disorders has not yielded consistent results. Some studies have found that living in a violent neighbourhood with high rates of homicide is a risk factor for development of mental disorders; most of these studies were conducted with adolescent and young adults populations [45,46]. However, other studies failed to corroborate these findings [38,47]. In line with our results, Curry et al. [38] did not observe a direct path between neighbourhood levels of violent crime and depressive symptoms, but they found that experiencing violence in the neighbourhood were associated with depressive symptoms. Stockdale et al. [47] reported that individuals who lived in areas with high crime rates and were exposed to violence were more likely to experience depressive or anxiety disorders compared with their counterparts living in areas with lower crime rates; however, for those with no violence exposure, neighbourhood crime was not associated with higher rates of these disorders. One possible explanation for our findings is that homicide rates in this study were evaluated around the victim’s place of residence as opposed to the place of occurrence. As most of the participants live in highly urbanised areas, it is likely that some of these crimes were not known in their neighbourhood.

### Strengths and limitations of the study

Our study has several strengths, including the longitudinal design, the use of multiple domains of community violence exposure, and the use of a large sample. As far as we know, this is the first study of this nature carried out in Latin America.

Nevertheless, some limitations of the study must be noted. Our sample represents a relatively homogenous group of civil servants working at a university and guaranteed a permanent job. Unlike the Brazilian socioeconomic structure, participants of the study had a medium to high socioeconomic background and a higher level of schooling. This homogeneity would have decreased the variability of both predictors and outcome within weighting areas, which could at least partly explain the lack of association between rates of homicides at the contextual level and the occurrence/persistence of psychological distress. Another explanation, as already stressed above, is the fact that homicide rates in this study were evaluated at the location of the victim’s residence instead of the location of occurrence.

Another related issue is that studies based on occupational cohorts have potential limitations for generalizing their results to the general population as this selection strategy limits investigation of those extremely poor and the unemployed. However, our study aims at estimating the association between certain exposure variables and an outcome and not at estimating prevalence of the study exposures or outcomes. Therefore, for this matter, high internal validity is the crucial aspect to consider. Occupational samples, for instance, are known to minimize losses during follow-up, one of the main problems in longitudinal studies.

The lack of a validated measure for the assessment of community violence poses another important methodological challenge in this study, and it reflects, at least in part, a limitation of community-violence studies. Two reviews that evaluated the effect of community-violence exposure and mental health outcomes of children and adolescents pointed out this issue, emphasizing that the lack of consistency in the examination of community-violence. According to the authors, the main discrepancies stems from the different ways it is assessed among the studies examined, the number of instruments used to measure exposure to community violence and different time of recall the exposure. In addition, not all studies separated witnessing violence from the victimization itself, making comparisons across studies complicated [48,49].

The measurement of individual’s exposure to violence is another study limitation. Violence exposure was assessed by means of three separate questions, which allowed the distinction between direct and indirect violence, but could not capture other important features, such as the occurrence of domestic violence. This issue has a close relationship with another limitation of the study, namely the absence of distinction between men’s and women’s exposure to violence. In general, men are usually more exposed to acute community and women to chronic domestic violence [41,43,50]. However, our study variables did not allow us to distinguish these two forms of violence.

The option for the inclusion of alcohol consumption as a confounding variable in the analysis should be seen in light of the longitudinal study design of this study. In fact, the relationship of alcohol consumption with psychological distress is often bidirectional, and cross-sectional studies have difficulty in establishing the direction of this association. However, in this study, for “incidence of psychological distress”, the temporal sequence of events is clear, since alcohol consumption was assessed retrospectively in the baseline and prevalent cases of psychological stress have been removed from the analysis. For the analysis of “persistence of psychological distress”, we cannot rule out the possibility of mental disorders can also lead to initiation and chronicity of alcohol. Nevertheless, we chose to keep the consumption of alcohol as a potential confounding, since many studies show that alcohol is an independent risk factor for the chronicity of mental disorders.

Another related issue is that, theoretically, alcohol consumption might be considered both a confounder and an intermediate variable (i.e., in the causal pathway between violence and psychological distress). However, results from multivariate analysis do not support the hypothesis of mediation, since the inclusion of alcohol consumption in the models did not reduce the effect of violence on psychological distress. Conversely, when alcohol consumption is included, the association increased, suggesting that this covariate is a confounder in this context.

Finally, the use of the GHQ-12, a screening instrument, rather than a standardized clinical interview that would generate a formal psychiatric diagnosis may have underestimated the associations under study, as the GHQ-12 is more sensitive to recent changes in psychological function. This may have caused a higher number of false positives among the cases of psychological stress, including individuals with milder or transient symptoms of psychological disturbance.

## Conclusions

Results of this study highlight the importance of assessing multiple forms of violence in research. In this study, exposure to both direct and indirect violence was associated to the occurrence and persistence of psychological distress. Unfortunately, it was not possible to investigate the effect of different types of direct victimization, like community and domestic violence. Future studies should overcome this gap, including more refined instruments on domestic, community and other types of violence, helping policymakers appreciate the evidence of more complex relationships among different types of violence and their outcomes.

We recommend further research investigating outcomes related to comorbidity of mental disorders, especially since previous studies have shown that, particularly among the youngest, chronic exposure to violence is a major risk factor for severe mental disorders as well as substance and alcohol abuse. We also believe that future research must continue to investigate the impact of high rates of chronic community violence on the prevalence of mental disorders in those living in large urban centres.
